# Development and evaluation of the Andhra Pradesh Children and Parent Study Physical Activity Questionnaire (APCAPS-PAQ): a cross-sectional study

**DOI:** 10.1186/s12889-016-2706-9

**Published:** 2016-01-19

**Authors:** Mika Matsuzaki, Ruth Sullivan, Ulf Ekelund, KV Radha Krishna, Bharati Kulkarni, Tim Collier, Yoav Ben-Shlomo, Sanjay Kinra, Hannah Kuper

**Affiliations:** 1 Department of Non-communicable Disease Epidemiology, London School of Hygiene and Tropical Medicine, Keppel Street, London, WC1E 7HT UK; 2 Department of Clinical Research, London School of Hygiene and Tropical Medicine, London, UK; 3 Department of Sport Medicine, Norwegian School of Sport Sciences, Oslo, Norway; 4 MRC Epidemiology Unit, University of Cambridge, Cambridge, UK; 5 National Institute of Nutrition, Hyderabad, India; 6 Institute of Health and Biomedical Innovation, Queensland University of Technology, Brisbane, Australia; 7 School of Social and Community Medicine, University of Bristol, Bristol, UK

**Keywords:** Health behavior, Physical activity, Developing countries, Survey methods

## Abstract

**Background:**

There is limited availability of context-specific physical activity questionnaires in low and middle income countries. The aim of this study was to develop and examine the validity of a new Indian physical activity questionnaire, the Andhra Pradesh Children and Parent Study Physical Activity Questionnaire (APCAPS-PAQ).

**Methods:**

The current study was conducted with the cohort from the Hyderabad DXA Study (*n* = 2321), recruited in 2009-2010. Criterion validity (*n* = 245) was examined by comparing the APCAPS-PAQ to a combined heart rate and motion sensor worn for 8 days. Construct validity (*n* = 2321) was assessed with linear regression, comparing APCAPS-PAQ against BMI, percent body fat, and pulse rate.

**Results:**

The APCAPS-PAQ criterion validity was variable depending on the PA intensity groups (ρ = 0.26, 0.07, 0.39; к = 0.14, 0.04, 0.16 for sedentary, light, moderate/vigorous physical activity (MVPA) respectively). Sedentary and light intensity activities from the questionnaire were underestimated when compared to the criterion data while MVPA in APCAPS-PAQ was overestimated. Higher time spent in sedentary activity in APCAPS-PAQ was associated with higher BMI and percent body fat, suggesting construct validity.

**Conclusions:**

The APCAPS-PAQ validity is comparable to other physical activity questionnaires. This tool is able to assess sedentary behavior, moderate/vigorous activity and physical activity energy expenditure on a group level with reasonable validity. This new questionnaire may be used for ranking individuals according to their sedentary time and physical activity in southern India.

**Electronic supplementary material:**

The online version of this article (doi:10.1186/s12889-016-2706-9) contains supplementary material, which is available to authorized users.

## Background

Physical activity (PA) has a multitude of positive effects on cardiovascular and musculoskeletal health and non-communicable disease (NCD) prevention across all ages [1–3]. Much of the evidence for the benefits of PA is based on studies from higher income countries [4, 5].

While the prevalence of NCDs has been rising sharply in low and middle income countries (LMIC) like India, there is limited evidence on the effects of PA on health in LMIC, partially due to a lack of valid and reliable instruments to assess physical activity [6, 7]. Available instruments, such as the International Physical Activity Questionnaire (IPAQ) and Global Physical Activity Questionnaire (GPAQ) were designed as surveillance instruments to capture PA at a population level and are often too prescriptive in style to fully encompass socio-cultural differences, thus preventing collection of detailed information on country-specific activities across multiple domains [8–10]. An accurate and reliable instrument for assessing PA in LMIC can improve our understanding of the association between PA and health outcomes and inform preventive action in LMIC settings.

We have previously developed the Indian Migration Study Physical Activity Questionnaire (IMS- PAQ) as an alternative PA collection tool and demonstrated its stability and validity [11]; however, this questionnaire required high level of data cleaning because of the open-ended questions. The Andhra Pradesh Children and Parent Study Physical Activity Questionnaire (APCAPS-PAQ) was therefore developed from the IMS-PAQ in order to improve data collection and analysis of questionnaire-based PA data within an Indian population. The aim of the present study was to examine the validity of this new instrument using objectively measured physical activity as the criterion.

## Methods

### Ethical approval and consent

Ethics committee approval was obtained from the All India Institute of Medical Sciences Ethics Committee (reference number A-60/4/8/2004) and the London School of Hygiene and Tropical Medicine. Consent was sought from the factory managers for the Indian Migrant Study and from the community leaders in the villages for the APCAPS study. Informed written consent was collected from all participants. All participants diagnosed with potential medical conditions were referred for appropriate treatment.

### Study design

The Hyderabad Dual X-ray Absorptiometry (DXA) Study (HDS) combined cross-sectional data from two studies based in Hyderabad, India: 1) the second follow-up study of the Andhra Pradesh Children and Parent Study (APCAPS) [12] and 2) the first follow-up study of the Hyderabad arm of the Indian Migration Study (IMS) [13]. Detailed descriptions of these studies have previously been published [12, 13], but they are described in brief below. All participants in HDS underwent clinical examination at the National Institute of Nutrition (NIN) in Hyderabad, India, in 2009-2010.

The APCAPS was established through long term follow-up of the participants of the Hyderabad Nutrition Trial (HNT: 1987-1990) [12]. Two thousand six hundred one individuals who were born during, and therefore part of, the HNT were invited to participate in the second wave of data collection in the APCAPS (2009-2010). For the validation part of this study, in order to increase the sample size and broaden the sample population, the parents of participants from the APCAPS study and a convenience sample of the NIN employees were also included (*n* = 73).

The IMS was a cohort study examining rural to urban migrants and their spouses recruited from a factory in Hyderabad as well as their siblings who had remained in a rural area [13]. A total of 1726 participants, who had attended clinic as part of the baseline study of the IMS in 2005-2007 and on whom information was available, were eligible for inclusion within the HDS. The current analysis used data from the Hyderabad arm of the first follow-up study in 2009-2010 (*n* = 890 for construct validity, *n* = 67 for criterion validity). The flowchart describing the number of individuals recruited, enrolled, and included in the analyses is presented in Additional file 1.

A similar process to the protocol for testing the validity of the IMS-PAQ was applied for the APCAPS-PAQ [11].

#### Criterion validity

All participants within the HDS were asked whether they would like to take part in this section of the study. Those agreeing to participate initially underwent a medical check by the doctor for step-test eligibility. Participants who were pregnant, had undergone knee surgery within the last year, had a diagnosed knee or leg problem, diagnosed heart problem or those who the doctor thought may be at risk from undergoing the step-test for individually calibrating heart rate to work load were only included in free-living data collection. 245 participants were included in the analysis for criterion validity.

#### Construct validity

Construct validity of the APCAPS-PAQ was assessed against BMI, body fat percentages measured from DXA, and resting heart rate. All 2321 participants who completed the APCAPS-PAQ were included in this analysis.

### Measurements

#### Questionnaire

All participants completed an interviewer-administered quantitative physical activity questionnaire (APCAPS-PAQ; Additional file 2). Participants were asked to recall information about activities undertaken in the last week in the following main domains; work, travel, leisure (sports/games/exercise), household, and sedentary and sleep. Within each domain participants were asked about the participation in up to 11 specific activities (based on those frequently reported in the open-ended IMS-PAQ) and up to two open-ended activities. For each activity, the average amount of time spent on the activity and the frequency of the activity were documented. Additionally, questions regarding demographic information, standard of living, and diet were collected in the main questionnaire.

Metabolic equivalent unit values (MET) were assigned to each activity using the Compendium of Physical Activity and WHO/FAO/UN guidelines [14, 15], supplemented with country specific values [16]. One MET is equivalent to resting metabolic rate of approximately 3.5 mL of O_2_/kg/min, or 1 kcal/kg/hour, corresponding to the resting metabolic rate of sitting quietly. Total activity was calculated as total MET (hr/day) by summing daily MET values of all activities. For occupational activities considered ‘more strenuous than walking,’ the Integrated Energy Index (IEI) was applied to correct total MET [17]. Duration of PA for different activity intensity categories from the questionnaire were calculated using previously published intensity thresholds; sedentary < 1.5 MET; light 1.5 to 3 MET; moderate 3 to 6 MET; vigorous > 6 MET [3]. As only 3 % of the sample reported participation in vigorous activity, moderate and vigorous activity was subsequently regrouped as moderate-and-vigorous physical activity (MVPA). Physical activity energy expenditure (PAEE) for PAQ was calculated as Total Activity MET (hr-day) minus MET hr-day for sleep and MET hr-day equivalent of resting energy expenditure (REE) while being awake, multiplied by 4.183 to give kj/kg/day.

Forty nine participants (2 %) were excluded from analysis: 39 (1.6 %) were unable to recall more than 12 h of activity a day, one person recalled more than 36 h of activity, two people had a Total Activity value <26 MET hrs-day and one person had a Total Activity value >70 MET hrs-day. A total of 2,321 participants were included for the construct validity section.

#### Anthropometric data

Weight was measured to the nearest 0.1 kg with digital weighing machine (Seca 899) and standing height to the nearest 1 mm with a plastic stadiometer (Leicester height measure; supplied by Chasmors, London). Waist circumference (WC) was measured to the nearest mm using a non-stretch metal tape at the narrowest point of the abdomen between the ribs and the iliac crest at the end of expiration and hip circumference at the widest part of the buttock. Each measure was assessed twice and the average was used in the analysis. Body mass index (BMI) was calculated by dividing weight (kg) by height^2^ (m^2^).

#### Dual energy x-ray

Participants underwent whole body DXA scans on a Hologic DXA machine (91 % on Discovery A model and 9 % on QDR 4500 Elite) to provide measures of total body fat (g) and total body fat percentage (%). Whole body scans were visually checked for artifacts and those with major artifacts such as movements were removed from the analyses.

#### Actiheart

A combined heart rate and motion sensor device (Actiheart, CamNtech, UK) was used to test criterion validity of the APCAPS-PAQ. Actiheart records heart rate (HR), inter-beat-interval (IBI), and body movement by accelerometry. The Actiheart data collection consisted of three phases: signal test, step-test and free-living data set-up.

The combined sensor was attached with electrode pads (Red Dot 2570, 3 M) to participants chest [18]. Participants eligible for the step test underwent a brief practice test to ensure familiarity with the procedure. The monitors were initialized to record step-test data at a pre-determined time (usually 2-4 min from time of initializing). The step-test consisted of a maximum of eight-minutes of stepping onto and off a step of a set height of 200 mm. The speed of stepping increased from 15 to 33 steps per minute. At the end of the 8 min, the participant was asked to sit down and refrain from talking or moving for 2 min whilst their recovery HR was recorded.

After step-test completion, the monitor was set up to collect data for free-living activity. All monitors were initialized to record data for ≤30 s epochs for a minimum of 8 days. Participants were reminded that the monitors were to be worn at all times including sleep and bathing. Motion sensor data was expressed in counts per minute (cpm) and HR in beats per minute (bpm). These data were used to estimate PAEE (kj/kg/day) using the combined output of the acceleration and heart rate signals based on individual calibration from step-test in a branched equation model [19, 20]. Time (min/d) spent in different activity intensities (sedentary, light, MVPA) was estimated from combined sensing [21]. Periods of non-wear are inferred from the combination of non-physiological heart rate and prolonged periods of inactivity, which are taken into account to minimise diurnal information bias when summarising the intensity time-series into PAEE (kJ/kg/day) and time spent in sedentary, light, and MVPA (min/day).

Seventeen participants did not complete a step-test; for those participants, group calibration values were applied to calculate PAEE and time spent in different intensities [21].

### Statistical analysis

Socio-demographic characteristics are presented as means and standard deviation (SD) or frequency and percentage (%). PA characteristics are means and 95 % confidence intervals (95 % CI) except for variables with positively skewed distributions where geometric mean and 95 % CI are reported.

Criterion validity was assessed with Spearman rank correlation (ρ) by comparing PAEE and time spent in different intensity categories from the combined sensor to that obtained by the questionnaire. Sensitivity analysis was run to examine correlation, agreement and bias between the questionnaire-based and objectively measured PAEE and time spent in light and MVPA intensity, using a MET value of 2.0 for walking, which was originally assigned a MET-value of 3.5. This was applied to identify what effect the most commonly reported PA had on criterion validity within this population.

Mean bias was calculated by subtracting objectively measured PA from PA estimated from APCAPS-PAQ (i.e. PAEE, light and MVPA). The Bland-Altman method was used to investigate evidence of systematic bias between the APCAPS-PAQ and the criterion instrument.

Construct validity was estimated by fitting linear regression models to the data to identify the relationship between tertiles of activity intensity (sedentary, light, MVPA) as measured by the APCAPS-PAQ and BMI and percent body fat. Tertiles for each intensity category were produced with the lowest tertile representing the reference group. Model 1 adjusted for age and sex and accounted for the clustered nature of the data (sibling-pairs) while Model 2 additionally adjusted for time spent in other activity intensities.

All data were analyzed using stata 11 for windows software.

### Quality assurance

All protocols and equipment were first pilot-tested. Fieldworkers underwent training and standardization at the outset and subsequently every 6 months. Anthropometric instruments were calibrated at the start of each clinic session. A spine phantom was scanned on DXA every day to check for acceptable ranges. A single DXA technician scanned all participants. The calibration factor of each combined heart rate and movement monitor was checked to ensure that it was within range. If the calibration factor was determined to be erroneous, data for this file was excluded from further analysis.

## Results

We invited 4327 people (1726 from IMS and 2601 from APCAPS) to attend the clinic at the National Institute of Nutrition and examined 2370 (55 %); 918 from IMS (53 %) and 1446 from APCAPS (56 %). Among the IMS participants, clinic attendees did not differ in age from non-attendees (47.8 and 47.2 years, *p* = 0.15) or by percentage female (47 % and 48 %, *p* = 0.82). A higher proportion of clinic attendees were urban dwellers (61 % and 47 %, *p* <0.001). Among the APCAPS participants, clinic attendees were similar in age to non-attendees (20.1 and 20.2 years, *p* = 0.03) but were much more likely to be male than female (68 % and 28 %, *p* <0.001).

The characteristics of the total study population (*n* = 2321) are shown in Tables 1 and 2. The IMS participants were older and more likely to be women, had higher BMI, percentage body fat, and blood pressure than the APCAPS participants. Sedentary behavior was the most frequently reported activity, accounting for 409 min/day (95 % CI: 407 to 424). The least reported activity intensity was MVPA. This pattern held true for women when stratified by sex but men reported roughly 30 min/day more in light activity (398 min/day; 95 % CI: 385 to 403) compared to sedentary activity (364 min/day; 95 % CI: 360 to 380). The population spent an average of 1 h 46 min/day watching television, with women reporting over 2 h/day compared to 1 h 37 min/day for men. Total activity was higher in men (39.6 MET hrs-day), than women (36.0 MET hrs-day).

**Table 1:** Characteristics of the Hyderabad DXA Study (HDS) participants by sub-sample type

	Construct validity			Criterion validity			
	All participants (*n* = 2,321)	IMS (*n* = 890)	APCAPS (*n* = 1,431)	All participants (*n* = 245)	IMS (*n* = 67)	APCAPS (*n* = 105)	APCAPS+ (*n* = 73)
Residence N, (%)							
Urban	545 (24)	545 (62)	-	78 (31)	64 (96)	-	14(19)
Rural	1,764 (76)	333 (38)	1,431 (100)	167 (68)	3 (4)	105 (100)	59(81)
Sex (%)							
Men	1,441 (62)	471 (53)	970 (68)	136 (56)	36 (54)	58 (55)	42(58)
Women	880 (38)	419 (47)	461 (32)	109 (44)	31 (46)	47 (45)	31(42)
Age (year)	31.1 (14.6)	48.3 (8.3)	20.3 (1.2)	35.0 (14.2)	48.0 (8.8)	21.5 (1.3)	42.3(11.8)
Anthropometry							
Weight (kg)	56.9 (12.6)	65.5 (11.9)	51.5 (9.7)	56.8 (12.9)	67.1 (10.6)	50.1 (9.1)	57.0(13.6)
Waist (cm)	75.4 (12.5)	86.5 (10.4)	68.2 (7.5)	75.2 (12.6)	87.6 (9.8)	66.9 (7.2)	75.8(11.6)
Height (cm)	160.9 (8.9)	159.2 (8.7)	162.1 (8.8)	159.4 (8.6)	158.9 (7.5)	160.7 (9.2)	157.9(8.6)
BMI (kg/m^2^)	22.0 (4.7)	25.9 (4.4)	19.5 (2.9)	22.3 (4.7)	26.6 (3.9)	19.3 (2.7)	22.7(4.4)
Percent body fat	21.7 (9.4)	30.5 (6.5)	16.5 (6.6)	23.4 (8.5)	31.7 (5.9)	17.7 (6.8)	24.3(6.0)

Data presented are means and standard deviation (SD) except for residence and sex which are frequency and percentage (%)

**Table 2:** Physical activity characteristics (mean and 95 % CI) of all HDS participants (*n* = 2,321) estimated from the Andhra Pradesh Children and Parents Study Physical Activity Questionnaire

	HDS total^a^ (*n* = 2,321)		
	Men (*n* = 1,441)	Women (*n* = 880)	Total (*n* = 2,321)
Total activity (MET hr/day)	39.6 (39.2, 39.8)	36.0 (35.6, 36.3)	38. 2 (37.9, 38.4)
Activity intensity (min/day)^b^			
Sedentary activity	364 (360, 380)	483 (477, 504)	409 (407, 424)
Light activity	398 (385, 403)	367 (350, 370)	386 (374, 388)
MVPA	171 (160, 174)	97 (53, 61)	113 (106, 116)
Television viewing (min/day)	97 (93, 101)	124 (116, 131)	106 (102, 109)

^a^This excludes 73 participants who were recruited only for the Actiheart portion of criterion validity test

Data in the table is presented as mean and 95 % confidence intervals (CI), except for moderate/vigorous physical activity (MVPA) and television viewing which are geometric means and 95 % CI

^b^Sedentary activity <1.5 METS, Light activity 1.5-3 METS, MVPA >3 METS.

### Criterion validity

Correlation between APCAPS-PAQ and the criterion method (Actiheart) values was higher for MVPA (*ρ* = 0.39, *p* <0.001) and PAEE (*ρ* = 0.37; *p* < 0.001) than for sedentary behavior (*ρ* = 0.26; *p* <0.001) (Table 3). No correlation was seen for time reported in light activity by the questionnaire and that measured by the Actiheart. Results for κ statistic followed a similar pattern although agreement values were lower. There was evidence for agreement between questionnaire-based (APCAPS-PAQ) and objectively measured PA across all PA variables for women but only for PAEE and MVPA in men.

**Table 3:** Correlation and к agreement reported in the APCAPS-PAQ and parameters derived from the Actiheart monitor for free-living activity in Indian adults

	Actiheart					
	All (*n* = 245)		Men (*n* = 136)		Women (*n* = 109)	
		p		p		p
PAEE (kj/kg/day)						
ρ	0.37	<0.001	0.24	0.006	0.46	<0.001
κ	0.21	<0.001	0.16	0.006	0.28	<0.001
Sedentary^a^						
ρ	0.26	<0.001	0.08	0.37	0.35	<0.001
κ	0.14	<0.001	0.04	0.25	0.27	<0.001
Light^a^						
ρ	0.07	0.30	−0.10	0.26	0.20	0.004
κ	0.04	0.21	−0.08	0.90	0.18	0.004
MVPA^a^						
ρ	0.39	<0.001	0.25	0.003	0.36	0.003
κ	0.16	0.004	0.15	0.02	0.16	<0.001

Data presented are: *ρ* (spearman rank correlation coefficient) and к coefficient

^a^Sedentary activity = time spent in activities <1.5 MET; light activity = time spent in activities 1.5-3 METS; MVPA = time spent in activities >3 MET

*p*-value for spearman rank and к coefficient is a test of independence between the two data measures

**Fig. 1:**
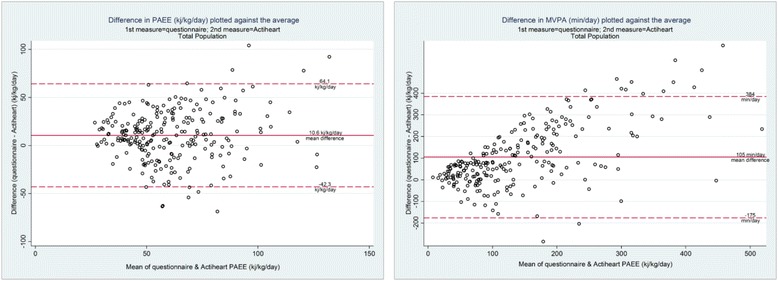
Bland-Altman plots of the difference vs. the mean of physical activity energy expenditure and moderate/vigorous physical activity reported in the APCAPS-PAQ and measured using the Actiheart

There was evidence of bias between mean PA values reported in the APCAPS-PAQ and those measured using the criterion method (Table 4). Bias was greatest for time spent on MVPA where the mean difference of 105 min/day equated to an overestimation of 76 % of the time recorded by combined heart rate and movement sensing. Proportionally, mean bias was smallest for time spent in sedentary behavior where the mean difference of -48 min/day equated to 6 % underestimation by the questionnaire compared with the criterion. Mean bias for PAEE (10.6 kj/kg/day*; p* <0.001) equated to an APCAPS-PAQ overestimation by approximately 16 %. Bland-Altman plots showed strong evidence of systematic bias in the over-reporting of activity within this population and indicate a greater degree of overestimation by the PAQ with a higher amount of time spent in MVPA (Fig. 1).

**Table 4:** Mean bias of the APCAPS-PAQ for physical activity measured in the APCAPS-PAQ and by the Actiheart in free-living adults

	All participants (*n* = 245)	Men (*n* = 136)	Women (*n* = 109)
PAEE kj/kg/day			
Questionnaire^a^	65.8 (25.8)	70.6 (26.2)	60.0 (24.1)
Mean bias^b^	10.6 (1.7)	9.9 (2.6)	11.4 (2.1)
p	<0.001	0.002	<0.001
Time spent in different activity intensities (min/day)			
Sedentary			
Questionnaire^a^	847 (206)	791 (188)	916 (207
Mean bias^b^	−48 (14.6)	−68 (20.4)	−23 (20.5)
p	0.001	0.001	0.26
Light			
Questionnaire^a^	395 (162)	418 (165)	366 (154)
Mean bias^b^	−63 (12.4)	−60 (17.7)	−66 (17.2)
p	<0.001	<0.001	<0.001
MVPA			
Questionnaire^a^	139 (151)	179 (145)	101 (152)
Mean bias^b^	105 (8.9)	118 (12.3)	89 (12.8)
p	<0.001	<0.001	<0.001

^a^Data from the questionnaire are mean and standard deviation (SD)

^b^Mean bias = questionnaire – Actiheart. Bias is reported as means and standard error (SE)

In the sensitivity analysis (substituting the MET value for walking from 3.5 to 2.0), the correlation between the APCAPS-PAQ and the criterion measure for time spent in light intensity activity became statistically significant (ρ = 0.14; *p* = 0.02) and κ agreement for MVPA increased from 0.16 to 0.30 (Additional file 3). Correlation and κ for PAEE and correlation for MVPA remained robust but decreased marginally. Sensitivity analysis increased correlation amongst men for PAEE and MVPA and κ agreement for PAEE. Amongst women, correlation and κ agreement increased for time spent in light activity and κ agreement for MVPA.

### Construct validity

**Fig. 2:**
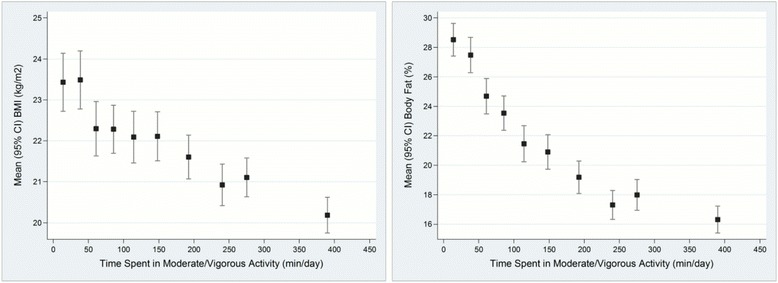
Unadjusted means and 95 % confidence intervals of body mass index and percent body fat for deciles of moderate/vigorous activity for the Hyderabad DXA Study (*n* = 2,321)

Table 5 and Fig. 2 show the association between PA variables and PA constructs. Total activity and time spent in different activity intensities was negatively associated with percentage body fat: Individuals in the highest tertile of total MET hrs-day had 0.89 % lower body fat (95 % CI -1.79 to -0.01; *p* <0.05) compared to individuals in the lowest tertile of total activity, a similar finding was seen for time spent in MVPA. Time spent in light and sedentary activity appeared to be most strongly associated with percentage body fat; participants in the highest tertile of light activity displayed 1.86 % higher body fat (95 % CI 1.09 to 2.61 %, *p* <0.001) and those in the highest tertile of sedentary activity had higher body fat of 1.52 % (95 % CI 0.68 to 2.35; *p* <0.001).

**Table 5:** Construct Validity (linear regression analysis) for all participants of the APCAPS-PAQ (*n* = 2,321)

	BMI (kg/m^2^) (*n* = 2,319)		Body fat % (*n* = 2,286)	
	Model 1 β (95 % CI)	Model 2 β (95 % CI)	Model 1 β (95 % CI)	Model 2 β (95 % CI)
Total activity^a^				
Total MET 1	Ref	Ref	Ref	Ref
Total MET 2	0.04 (-0.35, 0.42)	0.32 (-0.15, 0.79)	−0.26 (-0.79, 0.35)	−0.36 (-1.02, 0.29)
Total MET 3	−0.33 (-0.70, 0.04)	0.27 (-0.38, 0.92)	−1.45** (-1.97, -0.92)	−0.89* (-1.79, 0.01)
Physical activity intensity^b^				
Sedentary activity 1	Ref	Ref	Ref	Ref
Sedentary activity 2	−0.17 (-0.52, 0.19)	−0.08 (-0.49, 0.33)	0.35 (-0.16, 0.86)	0.28 (-0.27, 0.84)
Sedentary activity 3	0.30 (-0.70, 0.67)	0.81* (0.22, 1.40)	0.78* (0.25, 1.30)	1.52** (0.68, 2.35)
Light activity 1	Ref	Ref	Ref	Ref
Light activity 2	0.15 (-0.23, 0.52)	0.51* (0.08, 0.89)	0.53* (-0.04, 0.98)	1.09** (0.46, 1.59)
Light activity 3	0.33 (-0.04, 0.70)	0.97** (0.33, 1.38)	0.97** (0.02, 1.04)	1.86** (1.09, 2.61)
MVPA activity 1	Ref	Ref	Ref	Ref
MVPA activity 2	−0.32 (-0.74, 0.10)	−0.39 (-0.83, 0.11)	−0.18 (-0.76, 0.39)	−0.04 (-0.64, 0.57)
MVPA activity 3	−0.61** (-1.02, -0.20)	−0.17 (-0.68, 0.34)	−1.58** (-2.17, -0.99)	−0.80* (-1.54, -0.05)

Model 1: Adjusting for age and sex and clustering (sibling-pairs). Model 2: Adjusting for age, sex and other physical activity participation (sedentary, light, MVPA) and clustering (sib-pair)

Sedentary activity = time spent in activities <1.5 MET; light activity = time spent in activities 1.5-3 METS; MVPA = time spent in activities >3 MET

^a^Categories reflect increasing total activity (MET hr/day), with category 1 as baseline (least activity)

^b^Categories reflect increasing time spent in specific activity intensity with category 1 as baseline (least time). *p* -values are from linear regression analyses * *p* < 0.05; ** *p* < 0.001

After adjusting for age, sex, migrant status and time spent in other PA intensities, the associations between total activity and time spent in different activity intensities with BMI were attenuated and became less consistent than those seen for percent body fat. Increased time spent in sedentary or light activity was associated with higher BMI; participants in the highest tertile of sedentary activity had increased BMI of 0.81 kg/m^2^ (95 % CI: 0.22 to 1.40) and those in the highest tertile of light activity had higher BMI of 0.97 kg/m^2^ (95 % CI: 0.33 to 1.38) compared to those in the lowest tertiles. There was no strong evidence for association between total activity and time spent in MVPA with BMI.

## Discussion

The Andhra Pradesh Children and Parent Study Physical Activity Questionnaire (APCAPS-PAQ) was developed in order to improve data collection and analysis of questionnaire-based PA data within an Indian population. The results presented here show that construct validity of the APCAPS-PAQ was fair (κ between 0.2 and 0.4 [22]). Increased physical activity levels were associated with decreased percent body fat and, to a lesser extent, BMI levels. The evidence for criterion validity was weaker and more variable by activity intensity and by sex.

The APCAPS-PAQ construct validity was comparable to those estimates produced for the IMS-PAQ [11]. Increased total activity and time spent in different activity intensities was negatively associated with percentage body fat, and to a lesser extent with BMI. Criterion validity within the APCAPS-PAQ also showed similar patterns to those seen within the IMS-PAQ as well as IPAQ [8, 11]. Validity coefficients were highest for MVPA and PAEE and lowest for time spent in sedentary behavior for the sample as a whole when compared with the criterion instrument. Time spent in light intensity activity did not show strong correlation or κ agreement for the sample as a whole or by sub-analysis except for women. These variations in agreement by activity intensity have been described in previous studies in South Africa and China [10]. The stronger correlations seen for women were in contrast to earlier reports [23]. Interestingly, the correlations were higher for men than women in criterion validity studies using accelerometers in IMS and HDS (data not shown) [11]. One reason for this difference may be because women in this community are engaged more in activities that require upper-body movement, which are poorly detected by accelerometers. The findings from the current criterion study suggests that the younger women in this community could recall activities more consistently and may be less prone to bias when reporting on the type of activity undertaken.

Participants overestimated PAEE and time spent in MVPA in the questionnaire but underestimated time spent in sedentary behavior and light intensity activity in the APCAPS-PAQ compared to the criterion instrument. Overestimation of PA levels has been acknowledged in other questionnaires like IPAQ as well [24]. When participants were asked additional probing questions after completing the standard IPAQ (e.g. “You said that you did vigorous physical activity on 2 days for an average of 2 h. Can you please tell me about that activity”), reported PA levels decreased [24]. Both a probing protocol and better training of interviewers may be able to reduce overreporting of the PA levels and improve the PA estimation [24]. Our findings also suggest that lowering the MET value for walking from 3.5 to 2.0 MET within an Indian population may improve the strength of PAQ validity, through mitigating systematic bias and overestimation.

### Strengths and limitations

The APCAPS-PAQ provided more region-specific detailed physical activity data than other available questionnaires. Our questionnaire predominantly consisted of closed questions, reducing under-reporting of activities by participants observed during the IMS-PAQ implementation, and reducing the time taken to administer the questionnaire and process the data. The HDS population was large and diverse, from both rural and urban areas of India and a range of ages. The inclusion of combined heart rate and movement sensing as the criterion instrument allowed us to address the potential problem associated with accelerometers, which are less accurate for assessing PA during specific activities such as walking while carrying loads, walking uphill, water based activities and bicycling: Further, hip mounted accelerometers are less accurate to detect all body movement, especially upper-body motion. Finally, the use of combined heart rate and movement sensing overcomes potential issues with non-wear time associated with accelerometry. Collecting physical activity data in a non-invasive way among both men and women within both urban and rural areas of India will help to inform and establish the dose–response relationship between PA and chronic disease within the country. Another strength was the use of DXA measures of body fat, which provided more accurate measures of body composition compared with anthropometric methods.

All questionnaires may be associated with recall and social desirability bias. However, a shorter time frame over the ‘last week’ may reduce recall bias compared with longer time frames. The response rate of 52 % for the IMS follow-up participants, who were wealthier and more educated than the national average, may have given rise to an issue of selection bias and as such, the physical activity may not be generalizable from this sub-sample. Another limitation is that the reliability of the questionnaire could not be determined as there was a wide variation in the time between repeated measurements among participants who agreed to return for the second measurement at later dates.

As the NCD epidemic continues in low and middle income countries, monitoring of physical activity levels becomes crucial to developing effective public health interventions. There is an urgent need to develop a reliable and valid PAQ in India as NCDs now account for 60 % of total death [25]. The current study suggests that the APCAPS-PAQ fills the niche for the Indian context, offering an India-specific tool that is moderately valid for ranking individuals based on reported physical activity. Further refinement may be needed for male participants and for the MET value of certain activities, such as walking and other light-intensity activities, within an Indian population. Additionally, this tool needs to be tested for reliability as well as validity in other settings in India before it can be scaled up more broadly. While international questionnaires such as the IPAQ and GPAQ have been validated in several countries, these questionnaires are limited in the breath of region-specific data [8, 10]. Analyses of detailed data across multiple domains of physical activities (i.e. sports, chores, farm work) collected in APCAPS-PAQ can augment the efforts to develop public health interventions that are more appropriate for the Indian context.

## Conclusions

The APCAPS-PAQ validity is comparable to other physical activity questionnaires. This tool is able to assess sedentary behavior, moderate/vigorous activity and physical activity energy expenditure on a group level with reasonable validity. This new questionnaire may be used for ranking individuals according to their sedentary time and physical activity in southern India.

## Additional files

Additional file 1: **A flowchart of recruitment and participation for criterion and construct validity studies for the Andhra Pradesh Children and Parents Study Physical Activity Questionnaire (APCAPS-PAQ) in 2009-2010.** (PDF 39 kb)

Additional file 2: **APCAPS-PAQ.** (DOCX 28 kb)

Additional file 3: **Sensitivity analysis for correlation and к agreement reported in the APCAPS-PAQ and parameters derived from the Actiheart monitor for free-living activity, assigning walking a MET value of 2.0.** (DOCX 13 kb)

## Abbreviations

APCAPS: Andhra Pradesh Children and Parents Study
BMI: body mass index
bpm: beats per minute
cpm: counts per minute
DXA: dual x-ray absorptiometry
GPAQ: Global Physical Activity Questionnaire
HDS: Hyderabad DXA Study
HR: heart rate
ICC: intraclass correlations
IEI: integrated energy index
IMS: Indian Migration Study
IPAQ: International Physical Activity Questionnaire
LMIC: low and middle income countries
MET: metabolic equivalent units
MVPA: moderate-to-vigorous activity
NCD: non-communicable disease
PA: physical activity
PAEE: physical activity energy expenditure
PAQ: physical activity questionnaire
REE: resting energy expenditure
WC: waist circumference
