# Supplementary material for: Development and evaluation of the Andhra Pradesh Children and Parent Study Physical Activity Questionnaire (APCAPS-PAQ): a cross-sectional study
Source: BMC Public Health. 2016 Jan 19;16:48. doi: 10.1186/s12889-016-2706-9 (PMC4717598; doi:10.1186/s12889-016-2706-9)
Supplement: Supplementary file 1 — A flowchart of recruitment and participation for criterion and construct validity studies for the Andhra Pradesh Children and Parents Study Physical Activity Questionnaire (APCAPS-PAQ) in 2009-2010. (PDF 39 kb) [file 12889_2016_2706_MOESM1_ESM.pdf]

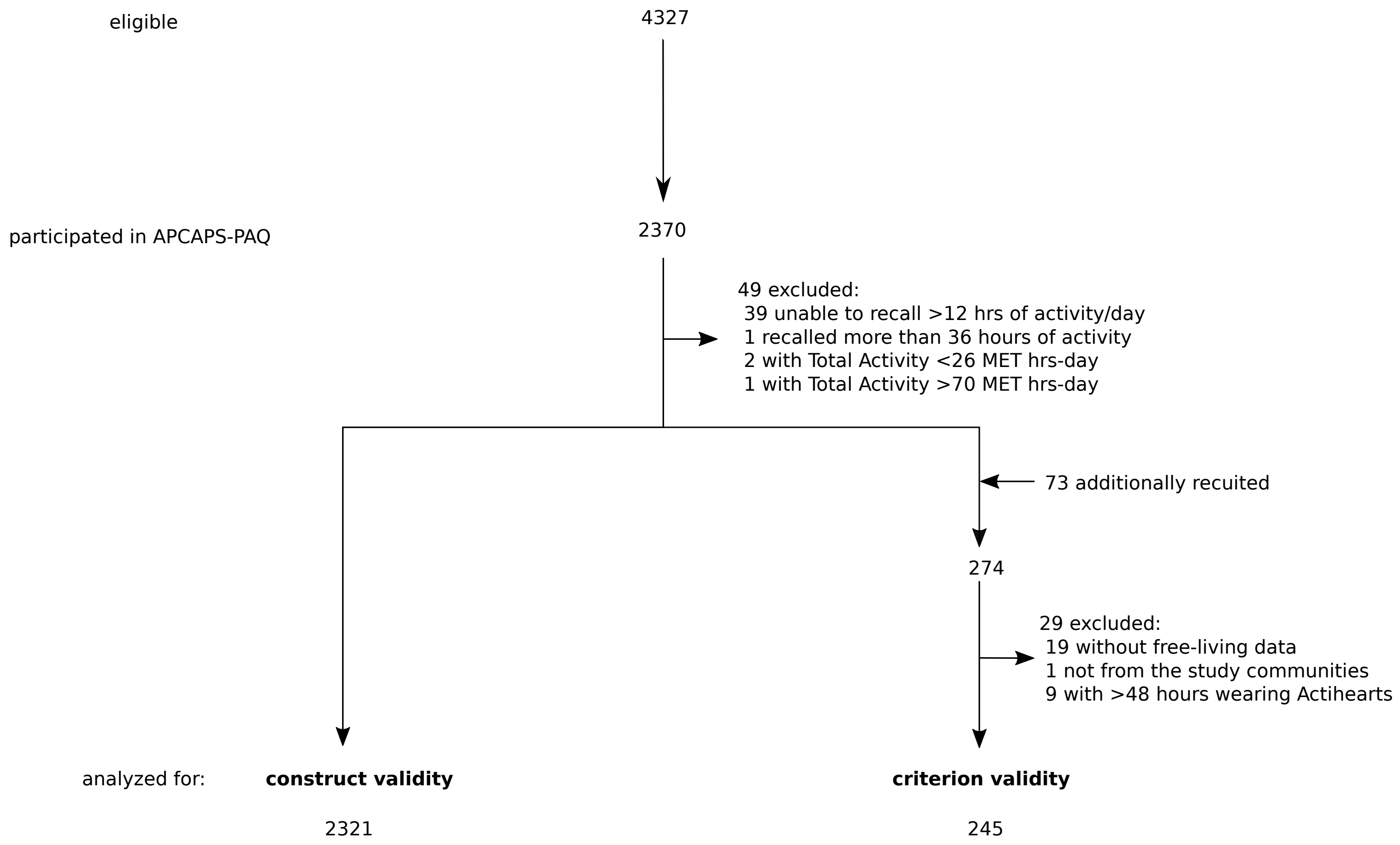

Supplemental Information S1: A flowchart of recruitment and participation for criterion and construct validity studies for the Andhra Pradesh Children and Parents Study Physical Activity Questionnaire (APCAPS-PAQ) in 2009-2010.
