# Supplementary material for: Development and evaluation of the Andhra Pradesh Children and Parent Study Physical Activity Questionnaire (APCAPS-PAQ): a cross-sectional study
Source: BMC Public Health. 2016 Jan 19;16:48. doi: 10.1186/s12889-016-2706-9 (PMC4717598; doi:10.1186/s12889-016-2706-9)
Supplement: Supplementary file 2 — APCAPS-PAQ. (DOCX 28 kb) [file 12889_2016_2706_MOESM2_ESM.docx]

**Supplemental Information 2 (S2): APCAPS-PAQ**

| *Now I am going to ask you questions about the time you spent doing different types of physical activity. Please recall the activities that you did in the* ***LAST WEEK.***  ***In case you travelled for a long duration to reach this place, or stayed back in this city for a few days, please recall the activities of the week before you left to this city.***  *The first questions are about your work/college. This includes paid jobs, working in your farm, study/training, any volunteer work or college activities.*  *Do not include unpaid work you might do around your home, like housework, garden work, and caring for your family. I will ask you about these later.* | | | | | | | | | | | | | |
| --- | --- | --- | --- | --- | --- | --- | --- | --- | --- | --- | --- | --- | --- |
| **Work related activity** | | | | |  | | | | | | | | |
| Do you currently have a job or do any unpaid work or study/training? Do not include household work, we will ask about this later. | | | | | | | | | | [1=Yes; 2=No]  **[IF NO, SKIP TO 11.8]** | | | |
| How many days did you work at the job or unpaid work in the last week? | | | | | | | | | | [In completed days] | | | |
| In the last week, how many hours per day did you spend at this work? | | | | | | | | | | .  [In completed half hours] | | | |
| **Of the hours you spend at work in a day during the last week I am going to ask you how many hours you spend in standing, sitting, walking and other strenuous activities (completed half hours):** | | | | | | | | | | | | | |
| (a) **Standing:** E.g. talk, lab work, supervise, mild cleaning, cattle grazing done standing. | | | | (b) **Sitting:** E.g. typing, computer work, cleaning grains, eating lunch, driving for your work, etc | | | | | (c) **Walking**: E.g. walking around, strolling, walking with light loads | | | | |
| . [hours] | | | | . [hours] | | | | | .[hours] | | | | |
| **If you spend any time at work on activities more strenuous than walking, please list these:** | | | | | | | | | | | | | |
|  | (i) Took part in this activity | | | | | | | (ii) Days per week | | | | | (iii) Total duration per day |
| (a) Carrying/walking with loads (15-25 kg) | [1=Yes; 2=No] | | | | | | | days | | | | | [mts] |
| (b) Carrying/walking with heavy load (≥25 kg) | [1=Yes; 2=No] | | | | | | | days | | | | | [mts] |
| (c) Lifting / loading of weights | [1=Yes; 2=No] | | | | | | | days | | | | | [mts] |
| (d) Pushing cart with a load | [1=Yes; 2=No] | | | | | | | days | | | | | [mts] |
| (e) Ploughing | [1=Yes; 2=No] | | | | | | | days | | | | | [mts] |
| (f) Digging | [1=Yes; 2=No] | | | | | | | days | | | | | [mts] |
| (g) Watering / weeding fields | [1=Yes; 2=No] | | | | | | | days | | | | | [mts] |
| (h) Cut / chop wood or stones | [1=Yes; 2=No] | | | | | | | days | | | | | [mts] |
| (i) Harvesting | [1=Yes; 2=No] | | | | | | | days | | | | | [mts] |
| (j) Any others? | [1=Yes; 2=No] | | | | | | |  | | | | |  |
| (k) _______________ |  | | | | | | | days | | | | | [mts] |
| (l) _______________ |  | | | | | | | days | | | | | [mts] |
| (m)_______________ |  | | | | | | | days | | | | | [mts] |
| **Travel to and from work**  *Now think about how you travelled to and from work over the LAST WEEK. Please do not include travelling activities if you have already mentioned while we discussed your work/college activities.* | | | | | | | | | | | | | |
|  | | | | | | (a) Days per week | | | | | | | (b) Total duration per day |
| During the last week, how many days did you travel on a motorised vehicle, like a car, bus, auto-rickshaw or motorcycle to and from work? | | | | | | days | | | | | | | [mts] |
| During the last week, on how many days did you cycle to and from work? | | | | | | days | | | | | | | [mts] |
| During the last week, on how many days did you walk to and from work? | | | | | | days | | | | | | | [mts] |
| **Travel apart from to and from work**  *Now think about how you travelled from place to place over the LAST WEEK, including places like stores, movies, visiting relatives etc but excluding to and from work. Please do not include travelling activities if you have already mentioned.* | | | | | | | | | | | | | |
|  | | | | | | | (a) Days per week | | | | (b) Total duration per day | | |
| During the last week, how many days did you travel to places on a motorised vehicle, like a car, bus, auto-rickshaw or motorcycle except to and from work? | | | | | | | days | | | | [mts] | | |
| During the last week, on how many days did you travel to places on a bicycle except to and from work? | | | | | | | days | | | | [mts] | | |
| During the last week, on how many days did you travel to places by walking except to and from work? | | | | | | | days | | | | [mts] | | |
| *Now I am going to ask you some questions about how you spent your time, apart from work outside of the home over the LAST WEEK* | | | | | | | | | | | | | |
| **Sports / games / exercise**  *Now think about all the physical activities that you did in the last 7 days solely for sport, exercise of leisure. Please do not include any activities you have already mentioned.* | | | | | | | | | | | | | |
| Name of activity | | (i) Took part in this activity | | | | | (ii) Days per week | | | | | (iii) Total duration per day | |
| (a) Walking normal speed for leisure | | [1=Yes; 2=No] | | | | | days | | | | | [mts] | |
| (b) Walking brisk speed for leisure | | [1=Yes; 2=No] | | | | | days | | | | | [mts] | |
| (c) Jogging/Running | | [1=Yes; 2=No] | | | | | days | | | | | [mts] | |
| (d) Badminton | | [1=Yes; 2=No] | | | | | days | | | | | [mts] | |
| (e) Cricket | | [1=Yes; 2=No] | | | | | days | | | | | [mts] | |
| (f) Yoga | | [1=Yes; 2=No] | | | | | days | | | | | [mts] | |
| (g) Swimming | | [1=Yes; 2=No] | | | | | days | | | | | [mts] | |
| (h) Football | | [1=Yes; 2=No] | | | | | days | | | | | [mts] | |
| (i) Volleyball | | [1=Yes; 2=No] | | | | | days | | | | | [mts] | |
| (j) Kabbadi | | [1=Yes; 2=No] | | | | | days | | | | | [mts] | |
| (k) Cycling | | [1=Yes; 2=No] | | | | | days | | | | | [mts] | |
| (l) Khokho | | [1=Yes; 2=No] | | | | | days | | | | | [mts] | |
| (m) Any others? | | [1=Yes; 2=No] | | | | |  | | | | |  | |
| (n) | |  | | | | | days | | | | | [mts] | |
| (o) | |  | | | | | days | | | | | [mts] | |
| (p) | |  | | | | | days | | | | | [mts] | |
| **Household activities**  *Now think about activities you do at home such as housework, gardening and hobbies. Please do not include any activities already mentioned.* | | | | | | | | | | | | | |
| Name of activity | | | (i) Took part in this activity | | | | (ii) Days per week | | | | | (iii) Total duration per day | |
| (a) Cooking | | | [1=Yes; 2=No] | | | | days | | | | | [mts] | |
| (b) Washing vessels | | | [1=Yes; 2=No] | | | | days | | | | | [mts] | |
| (c) Mopping | | | [1=Yes; 2=No] | | | | days | | | | | [mts] | |
| (d) Sweeping | | | [1=Yes; 2=No] | | | | days | | | | | [mts] | |
| (e) Wash clothes manually | | | [1=Yes; 2=No] | | | | days | | | | | [mts] | |
| (f) Dusting / cleaning | | | [1=Yes; 2=No] | | | | days | | | | | [mts] | |
| (g) Ironing and folding clothes | | | [1=Yes; 2=No] | | | | days | | | | | [mts] | |
| (h) Child care | | | [1=Yes; 2=No] | | | | days | | | | | [mts] | |
| (i) Collecting  fuel/fodder/water | | | [1=Yes; 2=No] | | | | days | | | | | [mts] | |
| (j) Animal care | | | [1=Yes; 2=No] | | | | days | | | | | [mts] | |
| (k) Gardening | | | [1=Yes; 2=No] | | | | days | | | | | [mts] | |
| (l) Any others? | | | [1=Yes; 2=No | | | |  | | | | |  | |
| (m)___________________ | | |  | | | | days | | | | | [mts] | |
| (n)___________________ | | |  | | | | days | | | | | [mts] | |
| (o)___________________ | | |  | | | | days | | | | | [mts] | |

| **Sedentary activities**  *The last question is about time spent sitting in the last 7 days. Do not include time spent sitting at work Please do not include any activities already mentioned.* | | | |
| --- | --- | --- | --- |
| Name of activity | (i) Took part in this activity | (ii) Days per week | (iii) Total duration per day |
| (a) Reading for leisure | [1=Yes; 2=No] | days | [mts] |
| (b) Computer/computer games/internet for leisure | [1=Yes; 2=No] | days | [mts] |
| (c) Watching TV/ movies | [1=Yes; 2=No] | days | [mts] |
| (d) Indoor games (e.g. chess, carom, playing cards) | [1=Yes; 2=No] | days | [mts] |
| (e) Prayer/meditation | [1=Yes; 2=No] | days | [mts] |
| (f) Listening to music/radio | [1=Yes; 2=No] | days | [mts] |
| (g) Sewing /embroidery/ knitting | [1=Yes; 2=No] | days | [mts] |
| (h) Socialising (talking outside working hours or on phone) | [1=Yes; 2=No] | days | [mts] |
| (i) Any others? | [1=Yes; 2=No] | days | [mts] |
| (j)__________________ |  | days | [mts] |
| (k)__________________ |  | days | [mts] |
| (l)__________________ |  | days | [mts] |
