# Supplementary material for: Development and evaluation of the Andhra Pradesh Children and Parent Study Physical Activity Questionnaire (APCAPS-PAQ): a cross-sectional study
Source: BMC Public Health. 2016 Jan 19;16:48. doi: 10.1186/s12889-016-2706-9 (PMC4717598; doi:10.1186/s12889-016-2706-9)
Supplement: Supplementary file 3 — Sensitivity analysis for correlation and к agreement reported in the APCAPS-PAQ and parameters derived from the Actiheart monitor for free-living activity, assigning walking a MET value of 2.0. (DOCX 13 kb) [file 12889_2016_2706_MOESM3_ESM.docx]

Supplemental Information 3 (S3)

Table 6: Sensitivity analysis for correlation and к agreement reported in the APCAPS-PAQ and parameters derived from the Actiheart monitor for free-living activity, (assigning walking a MET value of 2.0)

|  |  | Actiheart Study | | | | | | | |  |
| --- | --- | --- | --- | --- | --- | --- | --- | --- | --- | --- |
|  |  | All participants  (n=245) | |  | Men  (n=136) | |  | Women  (n=109) | |  |
|  |  |  | p |  |  | p |  |  | p |  |
|  | | |  |  |  |  |  |  |  |  |
| PAEE ( kj/kg/day) | | |  |  |  |  |  |  |  |  |
| *p* |  | 0.35 | <0.001 |  | 0.31 | <0.001 |  | 0.38 | <0.001 |  |
| κ |  | 0.22 | <0.001 |  | 0.19 | <0.001 |  | 0.27 | <0.001 |  |
|  | | | | |  |  |  |  |  |  |
| Time spent in different activity Intensities (min/day) | | | | | |  |  |  |  |  |
| Light |  |  |  |  |  |  |  |  |  |  |
| *p* |  | 0.14 | 0.02 |  | -0.06 | 0.48 |  | 0.28 | 0.003 |  |
| κ |  | 0.04 | 0.22 |  | -0.09 | 0.93 |  | 0.20 | 0.002 |  |
|  |  |  |  |  |  |  |  |  |  |  |
| MVPA |  |  |  |  |  |  |  |  |  |  |
| *p* |  | 0.33 | <0.001 |  | 0.44 | <0.001 |  | 0.21 | 0.03 |  |
| κ |  | 0.30 | <0.001 |  | 0.13 | 0.05 |  | 0.22 | <0.001 |  |

Data presented are: *ρ*=spearman rank correlation coefficient and к coefficient.

Sedentary activity = time spent in activities <1.5 MET.

Light activity= time spent in activities 1.5-3 METS.

MVPA= time spent in activities >3 MET

P-value for spearman rank and к coefficient is a test of independence between the two data measures.

Values presented assume the activity walking has a MET value of 2.0
